# Association of Low to Moderate Alcohol Drinking With Cognitive Functions From Middle to Older Age Among US Adults

**DOI:** 10.1001/jamanetworkopen.2020.7922

**Published:** 2020-06-29

**Authors:** Ruiyuan Zhang, Luqi Shen, Toni Miles, Ye Shen, Jose Cordero, Yanling Qi, Lirong Liang, Changwei Li

**Affiliations:** 1 Department of Epidemiology and Biostatistics, University of Georgia College of Public Health, Athens; 2 Department of Health Care Administration, College of Health and Human Services, California State University, Long Beach; 3 Clinical Epidemiology and Tobacco Dependence Treatment Research Department, Beijing Institute of Respiratory Medicine, Beijing Chaoyang Hospital, Capital Medical University, Beijing, China

## Abstract

**Importance:**

Studies examining the association of low to moderate drinking with various cognitive functions have yielded mixed findings.

**Objective:**

To investigate whether associations exist between low to moderate alcohol drinking and cognitive function trajectories or rates of change in cognitive function from middle age to older age among US adults.

**Design, Setting, and Participants:**

A prospective cohort study of participants drawn from the Health and Retirement Study (HRS), a nationally representative sample of US adults, with mean (SD) follow-up of 9.1 (3.1) years. In total, 19 887 participants who had their cognitive functions measured in the HRS starting in 1996 through 2008 and who had participated in at least 3 biennial surveys were included. The data analysis was conducted from June to November 2019.

**Exposures:**

Alcohol consumption and aging.

**Main Outcomes and Measures:**

Trajectories and annual rates of change for the cognitive domains of mental status, word recall, and vocabulary and for the total cognitive score, which was the sum of the mental status and word recall scores. Participants were clustered into 2 cognitive function trajectories for each cognition measure assessed based on their scores at baseline and through at least 3 biennial surveys: a consistently low trajectory (representing low cognitive scores throughout the study period) and a consistently high trajectory (representing high cognitive scores throughout the study period).

**Results:**

The mean (SD) age of 19 887 participants was 61.8 (10.2) years, and the majority of the HRS participants were women (11 943 [60.1%]) and of white race/ethnicity (16 950 [85.2%]). Low to moderate drinking (<8 drinks per week for women and <15 drinks per week for men) was significantly associated with a consistently high cognitive function trajectory and a lower rate of cognitive decline. Compared with never drinkers, low to moderate drinkers were less likely to have a consistently low trajectory for total cognitive function (odds ratio [OR], 0.66; 95% CI, 0.59-0.74), mental status (OR, 0.71; 95% CI, 0.63-0.81), word recall (OR, 0.74; 95% CI, 0.69-0.80), and vocabulary (OR, 0.64; 95% CI, 0.56-0.74) (all *P* < .001). In addition, low to moderate drinking was associated with decreased annual rates of total cognitive function decline (β coefficient, 0.04; 95% CI, 0.02-0.07; *P* = .002), mental status (β coefficient, 0.02; 95% CI, 0.01-0.03; *P* = .002), word recall (β coefficient, 0.02; 95% CI, 0.01-0.04; *P* = .01), and vocabulary (β coefficient, 0.01; 95% CI, 0.00-0.03; *P* = .08). A significant racial/ethnic difference was observed for trajectories of mental status (*P* = .02 for interaction), in which low to moderate drinking was associated with lower odds of having a consistently low trajectory for white participants (OR, 0.65; 95% CI, 0.56-0.75) but not for black participants (OR, 1.02; 95% CI, 0.74-1.39). Finally, the dosage of alcohol consumed had a U-shaped association with all cognitive function domains for all participants, with an optimal dose of 10 to 14 drinks per week.

**Conclusions and relevance:**

These findings suggested that low to moderate alcohol drinking was associated with better global cognition scores, and these associations appeared stronger for white participants than for black participants. Studies examining the mechanisms underlying the association between alcohol drinking and cognition in middle-aged or older adults are needed.

## Key Points

**Question:**

Does an association exist between current low to moderate alcohol drinking and cognitive function trajectories or rates of cognitive decline from middle to older age among US adults?

**Findings:**

In this cohort study of 19 887 participants from the Health and Retirement Study, with a mean follow-up of 9.1 years, when compared with never drinking, low to moderate drinking was associated with significantly better trajectories of higher cognition scores for mental status, word recall, and vocabulary and with lower rates of decline in each of these cognition domains.

**Meaning:**

Current low to moderate alcohol consumption among middle-aged or older adults may be associated with better total cognitive function.

## Teaser

This cohort study assesses the associations between low to moderate alcohol drinking and cognitive function trajectories or rates of cognitive decline from middle to older age in a nationally representative sample of US adults.

## Introduction

Alcohol misuse is a leading cause of morbidity and mortality.^1^ Alcohol consumption is associated with a uniformly increased risk of hypertension and stroke, regardless of dose,^2^ and heavy and binge drinking is associated with a higher risk of cardiovascular disease.^3^ However, studies have also found that low to moderate alcohol consumption is associated with protective effects against cardiovascular diseases.^4,5,6^ Besides its role in physical health, low to moderate alcohol consumption has been shown to play a role in the development of cognitive impairment and dementia, conditions that are highly associated with cardiovascular diseases, although the findings are mixed. Specifically, some studies have reported benefits to cognitive function associated with low to moderate alcohol consumption,^7,8,9,10,11^ whereas others have found no, minimal, or even adverse effects associated with alcohol consumption.^12,13,14,15^ The Nurses’ Health Study^11^ followed up with approximately 11 000 participants for 2 years and found that moderate drinkers perform better on general cognitive function and verbal memory tests and have slower rates of decline with time on these 2 cognitive functions compared with nondrinkers. Similarly, a 10-year follow-up study among 7153 British civil servants^16^ identified a protective association between low alcohol consumption and rates of global cognitive function and executive function decline compared with never drinking among women but not among men. By contrast, the Whitehall II imaging substudy^15^ followed up with 550 participants for more than 30 years and found that even moderate drinking is associated with cognitive function decline.

Cognitive functions are affected by many factors and vary with time. A single measurement cannot capture all aspects of cognition function and thus decreases the statistical efficiency of identifying potential risk factors.^17,18,19^ Using repeated measurements at different follow-up times can produce a more reliable estimate for both interpopulation and intrapopulation variations of cognitive functions.^20^ Moreover, although cognitive functions may decline with age, this decline is heterogeneous among people in different age groups.^21,22^ Thus, this heterogeneity should also be considered in studies of cognitive function. A few studies focusing on the association of alcohol drinking with cognitive function decline have taken repeated measurements into account; however, those studies used net follow-up time, instead of age, to calculate the rates of cognitive function decline and did not consider the heterogeneous effects associated with age.^8,9,11,15^

The present study investigated the association of low to moderate alcohol consumption with cognitive functions by using repeated cognition measurements and evaluated the association of low to moderate alcohol consumption with age-related decline in cognitive function in a nationally representative sample of middle-aged and older US adults.

## Methods

### Study Population

The present study was a secondary analysis of data from the Health and Retirement Study (HRS), a longitudinal panel study that surveys a nationwide representative sample of about 20 000 middle-aged and older US adults. The HRS participants have been reexamined every 2 years since 1992 (HRS wave 1) to collect their health and economic information.^23^ The HRS initially used the Telephone Interview for Cognitive Status,^24^ a brief telephone screening system to assess cognitive functioning. Since 1996 (HRS wave 3), a modification of that survey has been used to measure cognitive functioning.^25^ For consistency in measurements of the different waves, the present analyses used data from wave 3 (1996) and later. Furthermore, we only included participants who participated in at least 3 biennial surveys. Our study followed the Strengthening the Reporting of Observational Studies in Epidemiology (STROBE) reporting guideline. This secondary analysis of deidentified data from the HRS was approved by the institutional review board of the University of Georgia at Athens. Written informed consent was obtained from all participants in the original HRS. No one received compensation or was offered any incentive for participating in this study.

### Cognitive Function Tests

The HRS used age to determine which cognitive tests would be administered. Specifically, all respondents older than 65 years received a full set of tests in and before 1998 (wave 4); all new respondents received a full set of tests regardless of their age after 1998; and all reinterviewed respondents younger than 65 years received 2 questions self-assessing their memory (present rating and the changes), immediate and delayed recall tests, a backward counting test, and a serial 7s subtraction test in which 7 is subtracted from a given number for 5 trials.^25^ Because the HRS recruited additional new participants for each wave of the survey, data collected for each participant’s entry into the HRS were treated as the baseline for that participant. The follow-up measurements were defined as measurements of onward waves for each participant.

Cognitive functioning was measured by assessing 3 domains: total word recall, mental status, and vocabulary. Total word recall was scored as the summed results of an immediate word recall test and a delayed word recall test and ranged from 0 to 20, reflecting the number of words that a participant could correctly recall immediately or 5 minutes after they were read a list of 10 words. Mental status was measured using a set of tests that assess knowledge, language, and orientation, with scores ranging from 0 to 15. Vocabulary, also known as crystalized intelligence, represents established knowledge and was tested by assessing the ability of the participants to provide the definitions of 5 given words, with scores ranging from 0 to 10. The total cognition score was calculated as the summed scores of the total word recall results and the mental status test results and ranged from 0 to 35.^25,26^ Higher cognition scores indicated better cognitive abilities.

### Alcohol Drinking and Covariates

Alcohol consumption was assessed using the following questions: “Have you ever drank any alcoholic beverages, such as beer, wine, or liquor?”; “In the last 3 months, on average, how many days per week have you had any alcohol to drink?”; and “In the last 3 months, on the days you drank, about how many drinks did you have?” After wave 3 of the HRS (1996), participants were initially assessed for ever drinking.^26^ Ever drinkers were further asked for their drinking status in the last 3 months. On the basis of the answers to those questions at baseline, we categorized HRS participants as never drinkers, former drinkers, or current drinkers. Former drinkers were defined as participants who drank alcohol more than 3 months before the baseline interview, and current drinkers were defined as participants who drank alcohol within 3 months before the interview.^26^ For current drinkers, we calculated the alcohol consumption as the product of the number of days of drinking per week and the number of drinks per day. Current drinkers were then further categorized as low to moderate drinkers or heavy drinkers. Women with 8 or more drinks per week or men with 15 or more drinks per week were categorized as heavy drinkers^27^; otherwise, current drinkers were defined as low to moderate drinkers. Other covariates included age, sex, race/ethnicity, years of education, marital status, tobacco smoking status, and body mass index. Tobacco smoking status was categorized into 3 groups: never smoker, former smoker, and current smoker based on self-reported responses to these 2 questions: “Have you ever smoked cigarettes?” and “Do you smoke cigarettes now?”

### Statistical Analysis

Baseline characteristics for all included participants and by cognitive function trajectories are presented as percentages for categorical variables and mean (SD) values or median values and interquartile ranges (IQRs) for continuous variables. Nonparametric tests, χ^2^ tests, or *t* tests were used to compare the distribution of those characteristics by trajectory groups for each cognitive function measure. Data analyses were performed using data from participants with complete observations.

Latent variable mixture modeling implemented using the SAS Proc Traj procedure was used to identify subgroups that shared a similar progression for an outcome over time; more specifically, a similar trajectory for the various cognitive functions tested during follow-up.^28,29^ We fitted all trajectory models in quadratic form and from a 1-trajectory group up to a 5-trajectory group. The bayesian information criterion and visual assessment in the balance of the number of participants in the trajectory groups were used to select the number of groups that best fit the data. Trajectory analyses were conducted for each cognition domain and for overall cognitive function. When 2 trajectory groups best fit the data, we further assessed the model fit for different forms, with the optimal model having 2 trajectory groups in cubic form for each cognitive function measure.

The annual rate of age-related change for each individual was calculated by regressing cognitive function traits with age using all observations that an individual contributed, and the coefficient of age was treated as the age-related annual rate of change.

Multivariate logistic and linear regressions were used to evaluate associations of alcohol drinking with cognitive function trajectories and age-related annual rate of change, respectively, after controlling for age, sex, race/ethnicity, educational level, marital status, smoking status, and body mass index. The association analyses were also conducted by sex and race/ethnicity. Sex and racial/ethnic differences were evaluated by adding an interaction term, that is, alcohol drinking by sex or alcohol drinking by race/ethnicity, respectively, to the fully adjusted model.

We further assessed the potential nonlinear association between the number of drinks per week and the odds ratio (OR) of being clustered into groups with lower cognitive performance by using a nonparametric method with restricted cubic splines after controlling for the same set of covariates as for the regression model.^30^ This method used the likelihood ratio method to test the nonlinearity between the model having only a linear association and the model having a linear association and cubic spline terms. Sensitivity analyses were performed after excluding participants with at least 1 chronic disease condition. The analyses were performed from June to November 2019 using SAS, version 9.4 (SAS Institute Inc), and R, version 3.5.1 (The R Foundation). Bonferroni corrections were applied to account for multiple testing, and a 2-side *P* = .01, correcting for 4 cognition measures, was considered a statistically significant association.

## Results

In total, 19 887 people who participated in the HRS between 1996 and 2008 were included in the present study study (eFigure 1 in the Supplement), and their mean (SD) follow-up was 9.1 (3.1) years. Total word recall was measured among all 19 887 HRS participants, mental status and total cognition score were evaluated among 12 683 (63.8%) participants, and vocabulary was assessed for 9931 (49.9%) participants. As shown in Table 1, the mean (SD) age of all study participants was 61.8 (10.2) years. The majority of the HRS participants were female (11 943 [60.1%]) and of white race/ethnicity (16 950 [85.2%]). In total, 10 824 (54.4%) participants were ever drinkers, among which, 3767 (18.9%) were former drinkers and 7057 (35.5%) were current drinkers. Most of the current drinkers (6010 [85.2%]) were low to moderate drinkers. In addition, among all participants, 7813 (39.5%) were former smokers, 3704 (18.7%) were current smokers, and 5596 (28.1%) had a marital status of single or separated. Overall, HRS participants had a mean (SD) of 12.4 (3.1) years of education and were overweight (mean [SD] body mass index calculated as weight in kilograms divided by height in meters squared, 27.3 [5.3]).

**Table 1.** Baseline Demographic Characteristics by Cognitive Function Score

Characteristic	No. (%) of participants								
	Overall	Total cognition score		Mental status		Word recall		Vocabulary	
	(n = 19 887)	Low (n = 3619)	High (n = 9064)	Low (n = 2972)	High (n = 9711)	Low (n = 9538)	High (n = 10 349)	Low (n = 2444)	High (n = 7487)
Age, mean (SD), y	61.8 (10.2)	67.7 (8.4)	67.2 (8.3)	67.9 (8.6)	67.2 (8.2)	61.8 (10.1)	61.8 (10.4)	70.0 (7.7)	69.6 (7.3)
Female	11 943 (60.1)	2055 (56.8)	5389 (59.5)	2035 (68.5)	5409 (55.7)	4930 (51.7)	7013 (67.8)	1455 (59.5)	4484 (59.9)
Black race/ethnicity	2937 (14.8)	1043 (28.8)	637 (7.0)	987 (33.2)	693 (7.1)	2051 (21.5)	886 (8.6)	760 (31.1)	500 (6.7)
Marital status of single or separated	5596 (28.1)	1429 (39.5)	2601 (28.7)	1297 (43.6)	2733 (28.1)	2857 (30.0)	2739 (26.5)	1034 (42.3)	2342 (31.3)
Educational level, mean (SD), y	12.4 (3.1)	9.8 (3.6)	12.9 (2.6)	9.5 (3.6)	12.8 (2.7)	11.3 (3.4)	13.4 (2.5)	9.4 (3.4)	12.8 (2.8)
Tobacco smoker									
Never	8269 (41.8)	1469 (40.8)	3844 (42.6)	1294 (43.8)	4019 (41.6)	3669 (38.7)	4600 (44.7)	1069 (44.0)	3159 (42.4)
Former	7813 (39.5)	1505 (41.8)	3996 (44.3)	1140 (38.6)	4361 (45.2)	3787 (39.9)	4026 (39.1)	1009 (41.5)	3405 (45.7)
Current	3704 (18.7)	623 (17.3)	1175 (13.0)	520 (17.6)	1278 (13.2)	2035 (21.4)	1669 (16.2)	352 (14.5)	883 (11.9)
Alcohol consumption									
Never	9063 (45.6)	2265 (62.6)	3981 (43.9)	1937 (65.2)	4309 (44.4)	4914 (51.5)	4149 (40.1)	1615 (66.1)	3362 (44.9)
Ever	10 824 (54.4)	1354 (37.4)	5083 (56.1)	1035 (34.8)	5402 (55.6)	4624 (48.4)	6200 (59.9)	829 (33.9)	4125 (55.1)
Former	3767 (18.9)	492 (13.6)	1634 (18.0)	416 (14.0)	1710 (17.6)	1606 (16.6)	2161 (20.9)	330 (13.5)	1331 (17.8)
Low to moderate	6010 (30.2)	719 (19.9)	3020 (33.3)	520 (17.5)	3219 (33.1)	2485 (26.1)	3525 (34.0)	436 (17.9)	2426 (32.4)
Heavy	1047 (5.3)	142 (3.9)	430 (4.7)	99 (3.3)	473 (4.9)	526 (5.5)	521 (5.0)	61 (2.5)	370 (4.9)
With a chronic disease	15 346 (77.2)	3161 (87.4)	7405 (81.2)	2610 (87.7)	7956 (82.0)	7619 (80.0)	7727 (74.5)	2138 (87.9)	6325 (84.3)
BMI, mean (SD)	27.3 (5.3)	27.4 (5.2)	26.6 (4.7)	27.5 (5.4)	26.7 (4.6)	27.8 (5.4)	26.9 (5.2)	27.3 (5.1)	26.5 (4.7)

Abbreviation: BMI, body mass index (calculated as weight in kilograms divided by height in meters squared).

**Figure 1.**
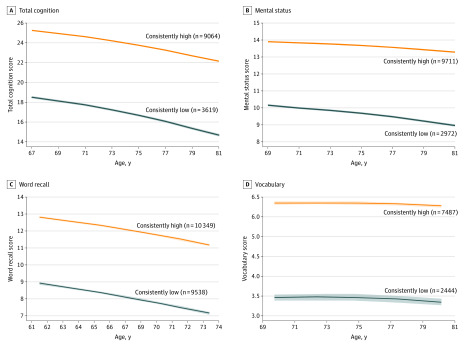
Trajectories of Cognition Scores From Baseline Through All Follow-up Assessments Shaded areas indicate upper and lower 95% CIs.

For each cognitive function measure, participants were categorized into a consistently low trajectory group (ie, cognitive test scores from baseline through follow-up were consistently low) or a consistently high trajectory group (ie, cognitive test scores from baseline through follow-up were consistently high) (Figure 1; eFigure 2 in the Supplement). Of 12 683 participants, 2972 (23.4%) had a consistently low trajectory for mental status, 9538 of 19 887 (48.0%) for word recall, 2444 of 9931 (24.6%) for vocabulary, and 3619 of 12 683 (28.5%) for total cognitive function score. As shown in Table 1, for each cognitive function measure, compared with participants in the consistently high trajectory group, those with a consistently low trajectory were more likely to be older, black individuals, single or separated, and tobacco smokers, and had fewer years of education and a higher body mass index. However, individuals with consistently low cognitive function results were less likely to be a current or former drinker (Table 1; eTable 1 in the Supplement).

**Figure 2.**
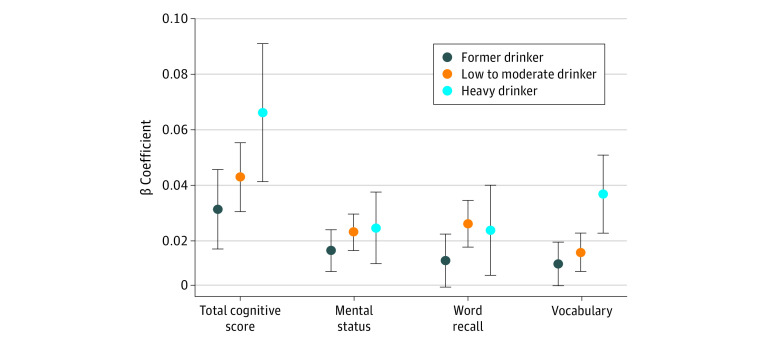
Association of Alcohol Consumption With the Annual Rate of Change in Cognition Scores Error bars indicate 95% CIs.

The associations of alcohol consumption and cognitive function trajectories are presented in Table 2. After controlling for all covariates, compared with never drinkers, current low to moderate drinkers were significantly less likely to be associated with consistently low trajectories for total cognitive score (OR, 0.66; 95% CI, 0.59-0.74), mental status (OR, 0.71; 95% CI, 0.63-0.81), word recall 0.74 (95% CI, 0.69-0.80), and vocabulary (OR, 0.64; 95% CI, 0.56-0.74) (all *P* < .001). Similarly, among former drinkers, ORs for being in the consistently low trajectory group were 0.72 (95% CI, 0.63-0.82) for the total cognitive score, 0.83 (95% CI, 0.72-0.95) for mental status, 0.76 (95% CI, 0.70-0.83) for word recall, and 0.73 (95% CI, 0.63-0.86) for vocabulary. Heavy drinkers had lower odds of being in the consistently low trajectory group only for the vocabulary test (OR, 0.51; 95% CI, 0.37-0.71). Low to moderate drinkers were also significantly associated with the age-related annual rate of change, with effect sizes of 0.04 (95% CI, 0.02-0.07; *P* = .002) for the total cognition score, 0.02 (95% CI, 0.01-0.03) for mental status, 0.02 (95% CI, 0.01-0.04; *P* = .01) for word recall, and 0.01 (95% CI, 0.00-0.03; *P* = .08) for vocabulary (Figure 2; eFigure 3 in the Supplement).

**Table 2.** Association of Alcohol Consumption With Consistently Low Trajectories on Measures of Cognitive Function^a^

Alcohol consumption	Total cognitive score		Mental status		Word recall		Vocabulary	
	OR (95% CI)	*P* value	OR (95% CI)	*P* value	OR (95% CI)	*P* value	OR (95% CI)	*P* value
Never	1 [Reference]		1 [Reference]		1 [Reference]		1 [Reference]	
Former	0.72 (0.63-0.82)	<.001	0.83 (0.72-0.95)	.008	0.76 (0.70-0.83)	<.001	0.73 (0.63-0.86)	<.001
Low to moderate	0.66 (0.59-0.74)	<.001	0.71 (0.63-0.81)	<.001	0.74 (0.69-0.80)	<.001	0.64 (0.56-0.74)	<.001
Heavy	0.87 (0.70-1.10)	.24	0.82 (0.64-1.06)	.13	1.02 (0.88-1.18)	.77	0.51 (0.37-0.71)	<.001

Abbreviation: OR, odds ratio.

^a^ Adjusted for age, sex, race/ethnicity, educational level, marital status, tobacco smoking, and body mass index.

Spline analyses showed significant U-shaped associations between weekly drinking doses and the odds of being in the consistently low trajectory group for all cognitive function domains (eFigure 4 in the Supplement). The weekly drinking dose at the turning points were 12 drinks for the total cognition score, 13 drinks for mental status, 10 drinks for word recall, and 14 drinks for vocabulary. Sensitivity analyses among participants with no chronic disease condition showed that the U-shaped association was still significant for the scores of total word recall (*P* = .001) and vocabulary (*P* = .004), but not for mental status (*P* = .88) or total cognition score (*P* = .19) (eFigure 5 in the Supplement).

The associations of alcohol drinking and cognitive functions were similar between men and women (eTable 2 in the Supplement) but differed for race/ethnicity. As shown in Table 3, low to moderate drinking was significantly associated with lower odds of having a consistently low trajectory for all 4 cognitive function measures only among white participants. For example, low to moderate drinking was associated with lower odds of having a consistently low mental status trajectory among white participants (OR, 0.65; 95% CI, 0.56-0.75) but not among black participants (OR, 1.02; 95% CI, 0.74-1.39) (*P* = .02 for interaction). In addition, the magnitude of the associations was stronger among white participants. The *P* values for the U-shaped associations were lower for white participants than for black participants and for men compared with women (eFigures 6, 7, 8, and 9 in the Supplement).

**Table 3.** Association Between Alcohol Drinking Status and Consistently Low Cognitive Function Trajectories Stratified by Race/Ethnicity^a^

Cognitive function	White participants			Black participants			*P* value for interaction
	No.	OR (95% CI)	*P* value	No.	OR (95% CI)	*P* value	
Total cognitive score							
Never drinker	5170	1 [Reference]	NA	1075	1 [Reference]	NA	
Former drinker	1860	0.70 (0.60-0.81)	<.001	266	0.83 (0.60-1.13)	.23	.56
Low to moderate drinker	3444	0.63 (0.56-0.71)	<.001	295	0.87 (0.63-1.18)	.37	.18
Heavy drinker	528	0.78 (0.61-1.00)	.05	44	2.20 (0.98-4.92)	.06	.02
Mental status							
Never drinker	5170	1 [Reference]	NA	1075	1 [Reference]	NA	
Former drinker	1860	0.80 (0.68-0.93)	.004	266	0.86 (0.63-1.17)	.34	.82
Low to moderate drinker	3444	0.65 (0.56-0.75)	<.001	295	1.02 (0.74-1.39)	.92	.02
Heavy drinker	528	0.76 (0.57-1.00)	.05	44	1.30 (0.64-2.65)	.46	.19
Word recall							
Never drinker	7355	1 [Reference]	NA	1706	1 [Reference]	NA	
Former drinker	3246	0.75 (0.68-0.82)	<.001	521	0.85 (0.68-1.07)	.17	.32
Low to moderate drinker	5402	0.72 (0.66-0.78)	<.001	608	0.89 (0.71-1.12)	.32	.09
Heavy drinker	945	0.98 (0.85-1.15)	.84	102	1.08 (0.64-1.80)	.78	.65
Vocabulary							
Never drinker	4143	1 [Reference]	NA	833	1 [Reference]	NA	
Former drinker	1465	0.66 (0.56-0.79)	<.001	196	1.04 (0.73-1.50)	.82	.04
Low to moderate drinker	2661	0.62 (0.53-0.72)	<.001	201	0.72 (0.49-1.04)	.08	.74
Heavy drinker	401	0.47 (0.33-0.67)	<.001	30	0.79 (0.33-1.87)	.59	.27

Abbreviations: NA, not applicable; OR, odds ratio.

^a^ Adjusted for age, sex, race, educational level, marital status, tobacco smoking, and body mass index.

## Discussion

The present study found that low to moderate drinking was associated with consistently high cognitive function trajectories, that is, cognitive test scores at the baseline middle-aged assessment were relatively high and remained high at each subsequent assessment, and a decreased rate of cognitive decline with age for middle-aged or older US adults. Alcohol consumption had a U-shaped relationship with cognitive function scores, with an optimal dosage of 10 to 14 drinks per week for all participants. The association of low to moderate drinking with higher cognitive function trajectories was stronger among white participants than among black participants.

Low to moderate alcohol drinking was associated with protecting cognitive function as assessed by the total cognitive score and the scores of each of the 3 cognition domains tested (mental status, word recall, and vocabulary). We also found that compared with never drinkers, low to moderate drinkers had slower rates of cognitive decline across time for all cognition domains evaluated. The association was strongest for the vocabulary test. These findings are in line with previous research. The Rancho Bernardo Study in southern California^31^ reported that moderate, regular alcohol drinking was associated with better cognitive function compared with never drinking among community-dwelling adults with a mean age of 73.2 years. The Nurses’ Health Study^11^ results suggested that up to 1 drink per day was associated with decreased risk of cognitive decline among women aged 70 to 81 years. Our study contributed further evidence that among a nationally representative sample of middle-aged or older adults, low to moderate drinking was associated with the protection of cognitive functions that may decrease with age. The association of low to moderate drinking with cognitive functions varies with age.^15,21,22^ The Whitehall II study of 550 participants in the UK^15^ reported that moderate drinkers with a mean age of 43 years were more likely to have hippocampal atrophy, and light drinking did not show a protective association compared with abstinence. However, that study defined moderate drinking as 14 to 21 units per week, which would be categorized as heavy drinking in our study. The association of low to moderate drinking with cognitive function in the younger age group warrants further investigation.

In the present study, although low to moderate drinking was associated with better cognitive functions and slower rates of cognitive decline, the associations between the weekly drinking dose and the various cognitive functions were U-shaped. The optimal alcohol dosage associated with better cognitive function was 10 to 14 drinks per week for all participants. Although the majority of drinkers in the HRS were low to moderate drinkers, 15.0% of white men (median 6; IQR, 2-12), 4.9% of white women (median, 3; IQR, 2-7), 15.7% of black men (median, 6; IQR, 2-10), and 5.6% of black women (median, 4; IQR, 2-6) had more than 14 drinks per week. Public health campaigns are still needed to further reduce alcohol drinking in middle-aged or older US adults, particularly among men. The mechanisms underlying the beneficial association of low to moderate alcohol consumption with cognitive function are unclear. The main hypotheses focus on cerebrovascular and cardiovascular pathways and on brain-derived neurotrophic factor. Several studies have found that low to moderate alcohol consumption is associated with better cardiovascular functions, fewer cardiac events, and longer survival compared with abstainers and heavy drinkers^4,5,6,32^; thus, the decreased risk of cognitive impairment has been thought to be associated with alcohol consumption. However, a recent study found that alcohol consumption increases the risk of hypertension and stroke regardless of dose,^2^ which decreases the likelihood of this potential mechanism. The role of alcohol drinking in cognitive function may be a balance of its beneficial and harmful effects on the cardiovascular system. Among low to moderate drinkers, the beneficial effects may outweigh the harmful effects on the cardiovascular system. Moderate drinking also increases brain-derived neurotrophic factor levels, a key regulator of neuronal plasticity and development, in the dorsal striatum, whereas levels of alcohol consumption leading to intoxication do not alter the mRNA expression levels of this factor.^33^

### Strengths and Limitations

Our study has several strengths. First, we analyzed data from a nationally representative, large sample of middle-aged or older US adults. Thus, findings of our study can be generalized to all middle-aged or older US adults. Second, repeatedly measured cognitive functions with a mean (SD) follow-up of 9.1 (3.1) years were used, which allowed us to estimate both long-term trajectories and age-associated annual rates of change in cognitive functions. Third, we used a group-based trajectory analysis approach to handle repeated measurements of the cognitive functions, which may eliminate random variations caused by a single measurement and thus provide higher accuracy of grouping and estimations.

However, certain limitations should also be acknowledged. First, alcohol consumption was self-reported, which could introduce recall bias that classifies heavy drinkers as low to moderate drinkers because participants tend to underestimate their alcohol consumption.^34^ Such misclassification would bias our association estimates toward the null, thus reducing statistical power to detect associations between alcohol drinking and cognitive functions. However, despite such a bias, our study still detected significant associations between alcohol consumption and cognitive function; thus, our data were sufficiently robust. Second, very few HRS participants had high weekly alcohol consumption, particularly among women and black participants, limiting the power of our analyses to identify an association of heavy drinking with cognitive function for these groups. Third, alcohol consumption tended to change with time; thus, this change may be associated with other factors that led to a change in cognitive function. Our study did not account for this possibility. Fourth, in the sensitivity analyses among participants with no chronic disease condition at baseline, the U-shaped associations were significant only for word recall and vocabulary, not for mental status and total cognitive score. The U-shaped associations for these latter 2 measures in the main analyses might have been due to confounding by health status. Healthy participants had higher cognitive function scores and might be engaged in more social activities and have higher alcohol consumption, leading to the higher cognitive function scores shown in the tails of the U-shaped plots. However, the study participants were middle-aged or older US adults, and 77.2% of the participants had at least 1 chronic disease condition. Therefore, the association between alcohol drinking and cognitive function may be applicable both to healthy people and to those with a chronic disease. Fifth, because fewer participants had high levels of weekly alcohol drinking, the 95% CIs for the risk of having low cognitive function among these people were wide. Thus, the reliability of the estimates for this group could be low.

## Conclusions

Our study suggested that low to moderate drinking was associated with better total cognitive function and better individual cognition domain results for word recall, mental status, and vocabulary among middle-aged or older men and women in the United States. Low to moderate alcohol use was also associated with slower rates of cognitive decline in those domains. These associations were stronger for white participants than for black participants. Furthermore, weekly alcohol consumption had U-shaped relationships with the cognitive functions assessed, with the strongest associations with better cognitive functions at a dosage of 10 to 14 drinks per week for all participants.

## Notes

Supplement. **eTable 1.** Baseline Statistics of the Study Population With *P* Values for Between Group Differences **eTable 2.** Associations of Drinking Status With Cognition Trajectories in Male and Female HRS Participants **eFigure 1.** Flowchart for Participants Selection and Cohort Construction **eFigure 2.** Cognition Trajectories (Mean Score) for 4 Cognitive Functions **eFigure 3.** Distribution of Annual Changing Rate in Different Drinking Groups **eFigure 4.** Nonlinear Relationship Between Weekly Drinking Level and Cognition Trajectory Among Current and Never Drinkers **eFigure 5.** Sensitivity Analyses for Nonlinear Relationship Between Weekly Drinking Level and Cognition Trajectory Among Healthy Current and Never Drinkers **eFigure 6.** Nonlinear Relationship Between Weekly Drinking Level and Cognition Trajectory Among Male Current and Never Drinkers **eFigure 7.** Nonlinear Relationship Between Weekly Drinking Level and Cognition Trajectory Among Female Current and Never Drinkers **eFigure 8.** Nonlinear Relationship Between Weekly Drinking Level and Cognition Trajectory Among White Current and Never Drinkers **eFigure 9.** Nonlinear Relationship Between Weekly Drinking Level and Cognition Trajectory Among African American (AA) Current and Never Drinkers
