# Supplementary material for: Association of Low to Moderate Alcohol Drinking With Cognitive Functions From Middle to Older Age Among US Adults
Source: JAMA Netw Open. 2020 Jun 29;3(6):e207922. doi: 10.1001/jamanetworkopen.2020.7922 (PMC7324954; doi:10.1001/jamanetworkopen.2020.7922)
Supplement: Supplement. — eTable 1. Baseline Statistics of the Study Population With P Values for Between Group Differences eTable 2. Associations of Drinking Status With Cognition Trajectories in Male and Female HRS Participants eFigure 1. Flowchart for Participants Selection and Cohort Construction eFigure 2. Cognition Trajectories (Mean Score) for 4 Cognitive Functions eFigure 3. Distribution of Annual Changing Rate in Different Drinking Groups eFigure 4. Nonlinear Relationship Between Weekly Drinking Level and Cognition Trajectory Among Current and Never Drinkers eFigure 5. Sensitivity Analyses for Nonlinear Relationship Between Weekly Drinking Level and Cognition Trajectory Among Healthy Current and Never Drinkers eFigure 6. Nonlinear Relationship Between Weekly Drinking Level and Cognition Trajectory Among Male Current and Never Drinkers eFigure 7. Nonlinear Relationship Between Weekly Drinking Level and Cognition Trajectory Among Female Current and Never Drinkers eFigure 8. Nonlinear Relationship Between Weekly Drinking Level and Cognition Trajectory Among White Current and Never Drinkers eFigure 9. Nonlinear Relationship Between Weekly Drinking Level and Cognition Trajectory Among African American (AA) Current and Never Drinkers [file jamanetwopen-3-e207922-s001.pdf]

# Supplementary Online Content

Zhang R, Shen L, Miles T, et al. Association of low to moderate alcohol drinking with cognitive functions from middle to older age among US adults. *JAMA Netw Open*. 2020;3(6):e207922. doi:10.1001/jamanetworkopen.2020.7922

**eTable 1.** Baseline Statistics of the Study Population With *P* Values for Between Group Differences

**eTable 2.** Associations of Drinking Status With Cognition Trajectories in Male and Female HRS Participants

**eFigure 1.** Flowchart for Participants Selection and Cohort Construction

**eFigure 2.** Cognition Trajectories (Mean Score) for 4 Cognitive Functions

**eFigure 3.** Distribution of Annual Changing Rate in Different Drinking Groups

**eFigure 4.** Nonlinear Relationship Between Weekly Drinking Level and Cognition Trajectory Among Current and Never Drinkers

**eFigure 5.** Sensitivity Analyses for Nonlinear Relationship Between Weekly Drinking Level and Cognition Trajectory Among Healthy Current and Never Drinkers

**eFigure 6.** Nonlinear Relationship Between Weekly Drinking Level and Cognition Trajectory Among Male Current and Never Drinkers

**eFigure 7.** Nonlinear Relationship Between Weekly Drinking Level and Cognition Trajectory Among Female Current and Never Drinkers

**eFigure 8.** Nonlinear Relationship Between Weekly Drinking Level and Cognition Trajectory Among White Current and Never Drinkers

**eFigure 9.** Nonlinear Relationship Between Weekly Drinking Level and Cognition Trajectory Among African American (AA) Current and Never Drinkers

This supplementary material has been provided by the authors to give readers additional information about their work.

© 2020 Zhang R et al. *JAMA Network Open*.

**eTable 1. Baseline Statistics of the Study Population With *P* Values for Between Group Differences**

| | | Overall | Total Cognition Score | | | Mental Status | | | Word Recall | | | Vocabulary | | |
|---|---|---|---|---|---|---|---|---|---|---|---|---|---|---|
| | | (n=19,887) | Low (n=3,619) | High (n=9,064) | p | Low (n=2,972) | High (n=9,711) | p | Low (n=9,538) | High (n=10,349) | p | Low (n=2,444) | High (n=7,487) | p |
| Age, mean (SD) | | 61.81 (10.24) | 67.75 (8.40) | 67.16 (8.26) | <0.001 | 67.89 (8.60) | 67.15 (8.21) | <0.001 | 61.82 (10.11) | 61.81 (10.35) | 0.959 | 70.04 (7.67) | 69.59 (7.29) | 0.008 |
| Female, N (%) | | 11943 (60.1) | 2055 (56.8) | 5389 (59.5) | 0.006 | 2035 (68.5) | 5409 (55.7) | <0.001 | 4930 (51.7) | 7013 (67.8) | <0.001 | 1455 (59.5) | 4484 (59.9) | 0.773 |
| Black, N (%) | | 2937 (14.8) | 1043 (28.8) | 637 ( 7.0) | <0.001 | 987 (33.2) | 693 ( 7.1) | <0.001 | 2051 (21.5) | 886 ( 8.6) | <0.001 | 760 (31.1) | 500 ( 6.7) | <0.001 |
| Single or Separated | | 5596 (28.1) | 1429 (39.5) | 2601 (28.7) | | 1297 (43.6) | 2733 (28.1) | | 2857 (30.0) | 2739 (26.5) | | 1034 (42.3) | 2342 (31.3) | <0.001 |
| Years of education, mean (SD) | | 12.40 (3.14) | 9.79 (3.57) | 12.94 (2.58) | <0.001 | 9.50 (3.56) | 12.82 (2.67) | <0.001 | 11.31 (3.35) | 13.40 (2.54) | <0.001 | 9.42 (3.35) | 12.79 (2.76) | <0.001 |
| Smoke Status, N (%) | | | | | <0.001 | | | <0.001 | | | <0.001 | | | <0.001 |
| | Never | 8269 (41.8) | 1469 (40.8) | 3844 (42.6) | | 1294 (43.8) | 4019 (41.6) | | 3669 (38.7) | 4600 (44.7) | | 1069 (44.0) | 3159 (42.4) | |
| | Former | 7813 (39.5) | 1505 (41.8) | 3996 (44.3) | | 1140 (38.6) | 4361 (45.2) | | 3787 (39.9) | 4026 (39.1) | | 1009 (41.5) | 3405 (45.7) | |
| | Current | 3704 (18.7) | 623 (17.3) | 1175 (13.0) | | 520 (17.6) | 1278 (13.2) | | 2035 (21.4) | 1669 (16.2) | | 352 (14.5) | 883 (11.9) | |
| Drinking status, N (%) | | | | | <0.001 | | | <0.001 | | | <0.001 | | | <0.001 |
| | Never | 9063 (45.6) | 2265 (62.6) | 3981 (43.9) | | 1937 (65.2) | 4309 (44.4) | | 4914 (51.5) | 4149 (40.1) | | 1615 (66.1) | 3362 (44.9) | |
| | Ever | 10824 (54.4) | 1354 (37.4) | 5083 (56.1) | | 1035 (34.8) | 5402 (55.6) | | 4624 (48.4) | 6200 (59.9) | | 829 (33.9) | 4125 (55.1) | |
| | Former | 3767 (18.9) | 492 (13.6) | 1634 (18.0) | | 416 (14.0) | 1710 (17.6) | | 1606 (16.8) | 2161 (20.9) | | 330 (13.5) | 1331 (17.8) | |
| | Current | 7057 (35.5) | 862 (23.8) | 3449 (38.1) | | 619 (20.8) | 3692 (38.0) | | 3018 (31.6) | 4039 (39.0) | | 499 (20.4) | 2794 (37.3) | |
| BMI, mean (SD) | | 27.34 (5.32) | 27.42 (5.21) | 26.64 (4.65) | <0.001 | 27.46 (5.42) | 26.69 (4.62) | <0.001 | 27.81 (5.44) | 26.91 (5.17) | <0.001 | 27.30 (5.05) | 26.50 (4.68) | <0.001 |

Note: BMI=body mass index; SD=standard deviation.

© 2020 Zhang R et al. *JAMA Network Open*.

## eTable 2. Associations of Drinking Status With Cognition Trajectories in Male and Female HRS Participants

| | | Male | | | Female | | | $P_{intx}$ |
|---|---|---|---|---|---|---|---|---|
| | | OR (95% CI) | P | | OR (95% CI) | P | | |
| TCS | | | | | | | | |
| | Never drinker | - | - | | - | - | | |
| | Former drinker | 0.81 (0.66 - 0.98) | 0.03 | | 0.66 (0.56 - 0.79) | <.0001 | | 0.09 |
| | Low to moderate drinker | 0.60 (0.51 - 0.71) | <.0001 | | 0.73 (0.62 - 0.86) | 0.0002 | | 0.26 |
| | Heavy drinker | 0.74 (0.54 - 1.01) | 0.06 | | 1.03 (0.75 - 1.42) | 0.86 | | 0.33 |
| MS | | | | | | | | |
| | Never drinker | - | - | | - | - | | |
| | Former drinker | 0.87 (0.69 - 1.11) | 0.27 | | 0.78 (0.66 - 0.93) | 0.005 | | 0.30 |
| | Low to moderate drinker | 0.61 (0.50 - 0.74) | <.0001 | | 0.77 (0.66 - 0.92) | 0.003 | | 0.16 |
| | Heavy drinker | 0.74 (0.50 - 1.09) | 0.13 | | 0.88 (0.63 - 1.24) | 0.48 | | 0.70 |
| WR | | | | | | | | |
| | Never drinker | - | - | | - | - | | |
| | Former drinker | 0.80 (0.69 - 0.93) | 0.003 | | 0.74 (0.67 - 0.83) | <.0001 | | 0.26 |
| | Low to moderate drinker | 0.72 (0.64 - 0.81) | <.0001 | | 0.76 (0.68 - 0.84) | <.0001 | | 0.86 |
| | Heavy drinker | 1.01 (0.81 - 1.25) | 0.93 | | 0.99 (0.81 - 1.21) | 0.93 | | 0.53 |
| VOC | | | | | | | | |
| | Never drinker | - | - | | - | - | | |
| | Former drinker | 0.75 (0.59 - 0.96) | 0.02 | | 0.70 (0.57 - 0.85) | 0.0004 | | 0.54 |
| | Low to moderate drinker | 0.65 (0.54 - 0.80) | <.0001 | | 0.63 (0.51 - 0.76) | <.0001 | | 0.68 |
| | Heavy drinker | 0.56 (0.36 - 0.86) | 0.01 | | 0.44 (0.27 - 0.72) | 0.0009 | | 0.35 |

Note: CI=confidence interval; OR=odds ratio; TCS=total cognitive score; MS=mental status; WR=word recall; VOC=vocabulary.

All analyses adjusted for age, gender, race, education, marital status, smoking status, and body mass index.

© 2020 Zhang R et al. *JAMA Network Open*.

## eFigure 1. Flowchart for Participants Selection and Cohort Construction

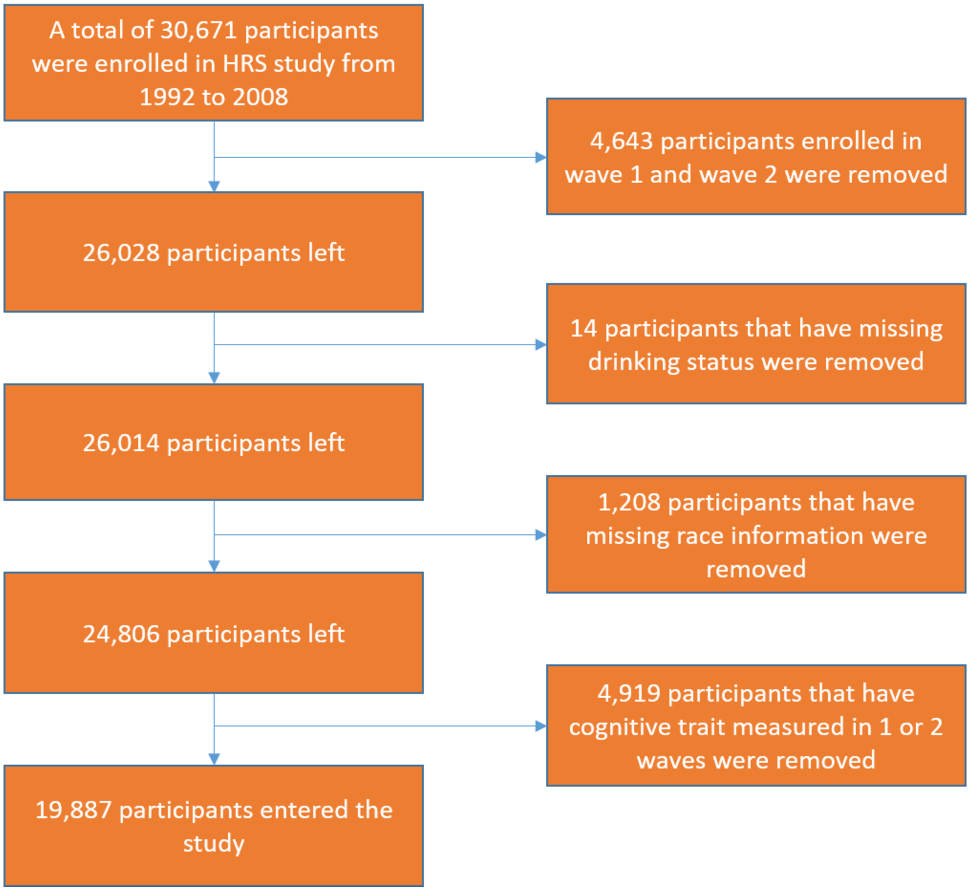


© 2020 Zhang R et al. *JAMA Network Open*.

## eFigure 2. Cognition Trajectories (Mean Score) for 4 Cognitive Functions

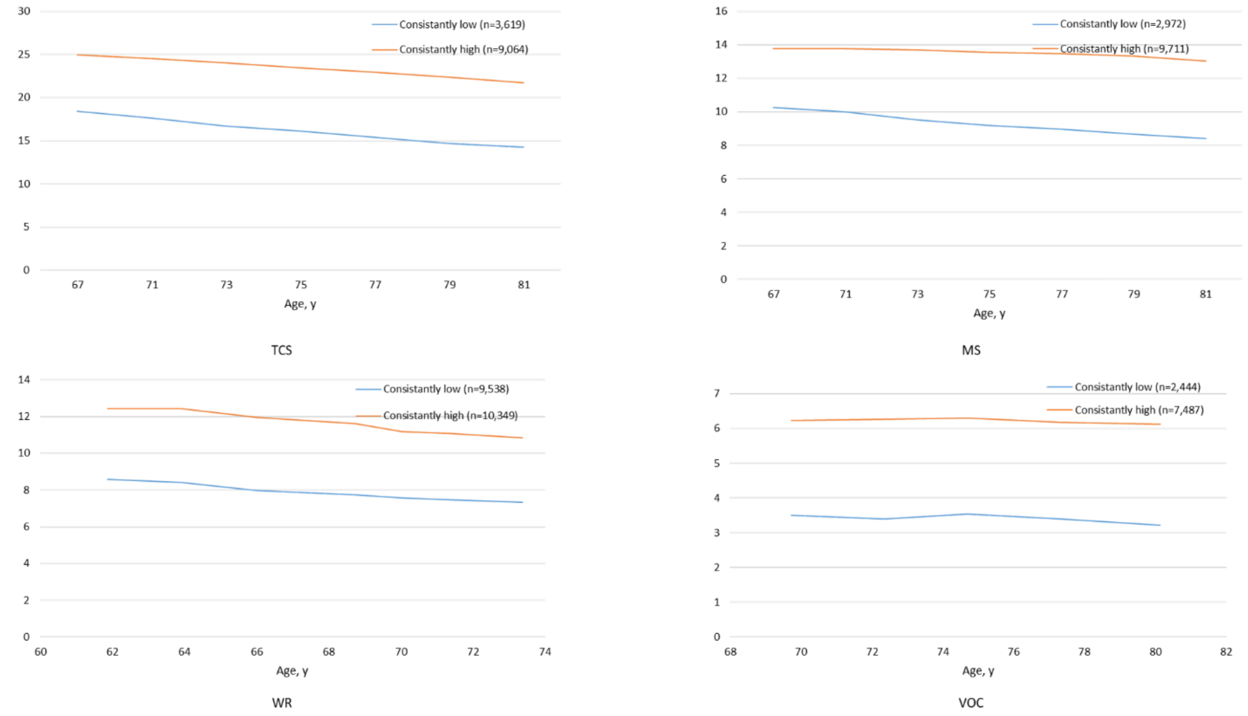


Note: TCS=total cognitive score; MS=mental status; WR=word recall; VOC=vocabulary; Group0=group with high cognitive function trajectory; Group1=group with low cognitive function trajectory.

© 2020 Zhang R et al. *JAMA Network Open*.

**eFigure 3. Distribution of Annual Changing Rate in Different Drinking Groups**

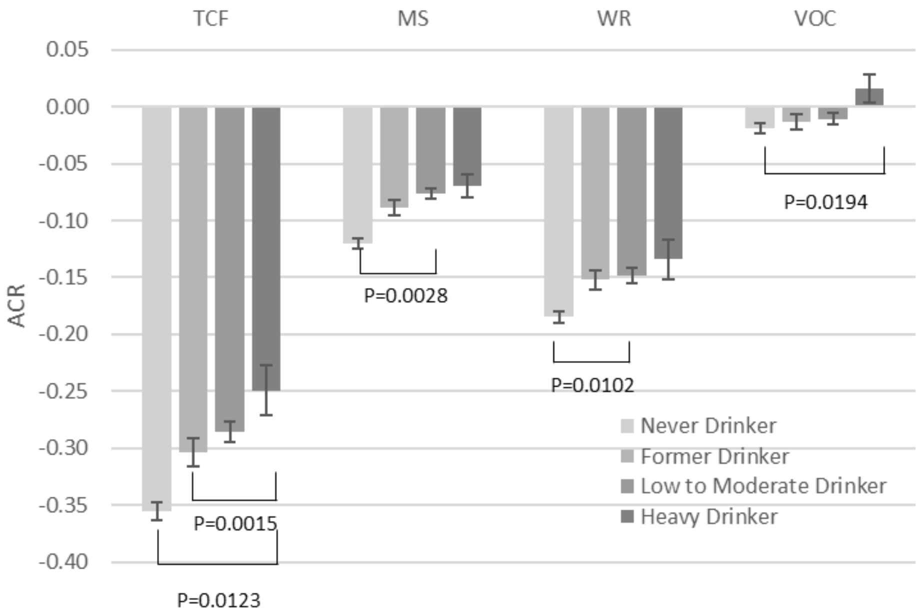


Note: Annual changing rate of TCS is significantly different between never drinker and heaver drinker, and former drinker and heavy drinker; annual changing rate of MS is significantly different between never drinker and low-to-moderate drinker; annual changing rate of WR is significantly different between never drinker and low-to-moderate drinker; annual changing rate of VOC is significantly different between never drinker and heavy drinker

© 2020 Zhang R et al. *JAMA Network Open*.

**eFigure 4. Nonlinear Relationship Between Weekly Drinking Level and Cognition Trajectory Among Current and Never Drinkers**

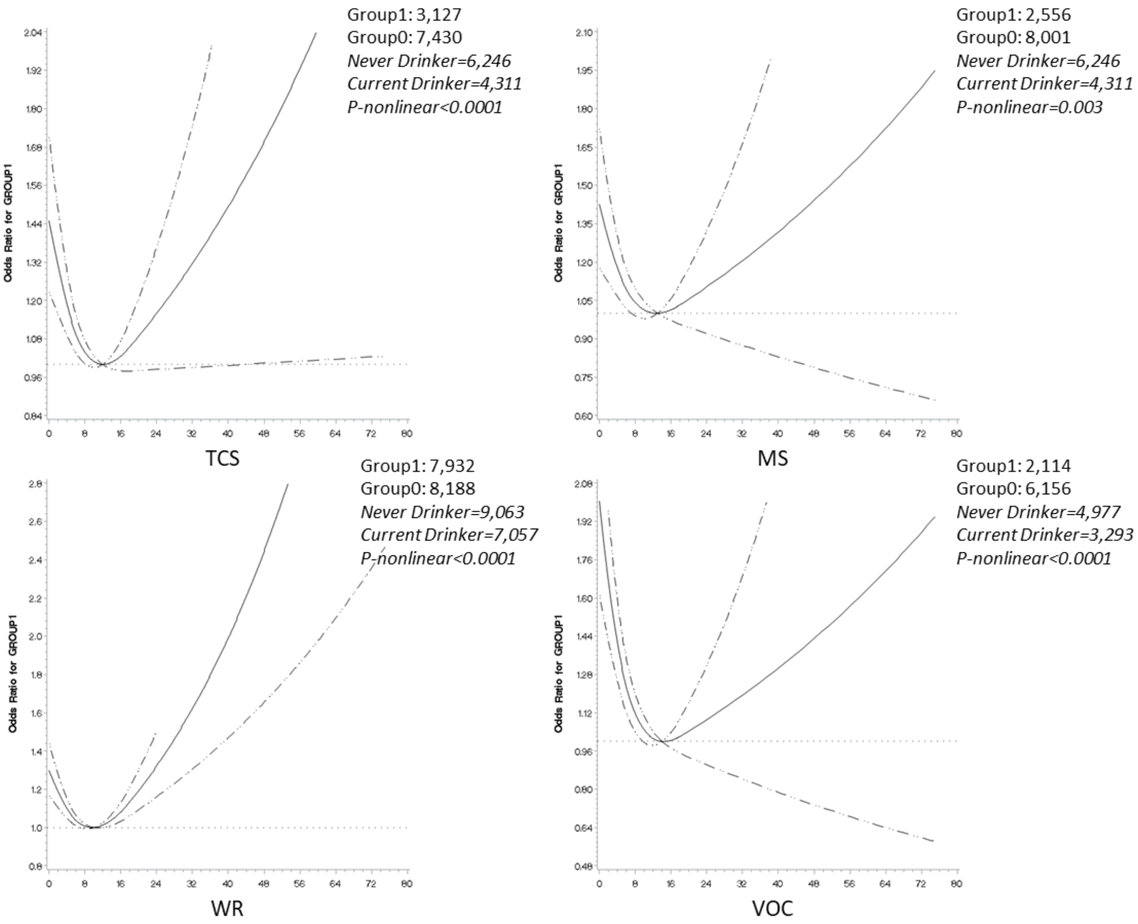


Note: Number of never drinker is 6246, 6246, 9063, and 4977; number of current drinker is 4311, 4311, 7057, and 3293 for TCS, MS, WR, and VOC, respectively.

© 2020 Zhang R et al. *JAMA Network Open*.

**eFigure 5. Sensitivity Analyses for Nonlinear Relationship Between Weekly Drinking Level and Cognition Trajectory Among Healthy Current and Never Drinkers**

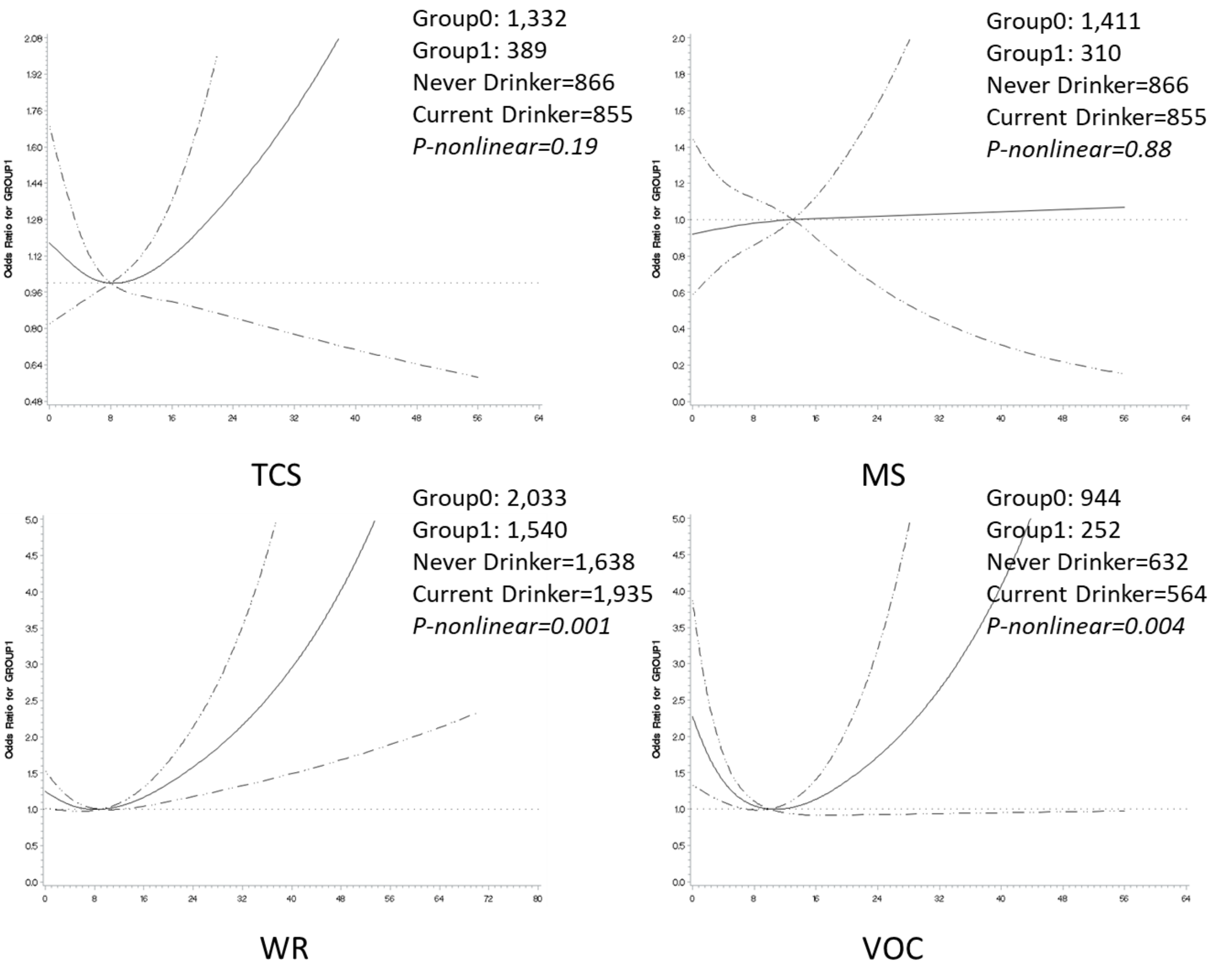


Note: Number of never drinker is 866, 866, 1638, and 632; number of current drinker is 855, 855, 1935, and 564 for TCS, MS, WR, and VOC, respectively.

© 2020 Zhang R et al. *JAMA Network Open*.

**eFigure 6. Nonlinear Relationship Between Weekly Drinking Level and Cognition Trajectory Among Male Current and Never Drinkers**

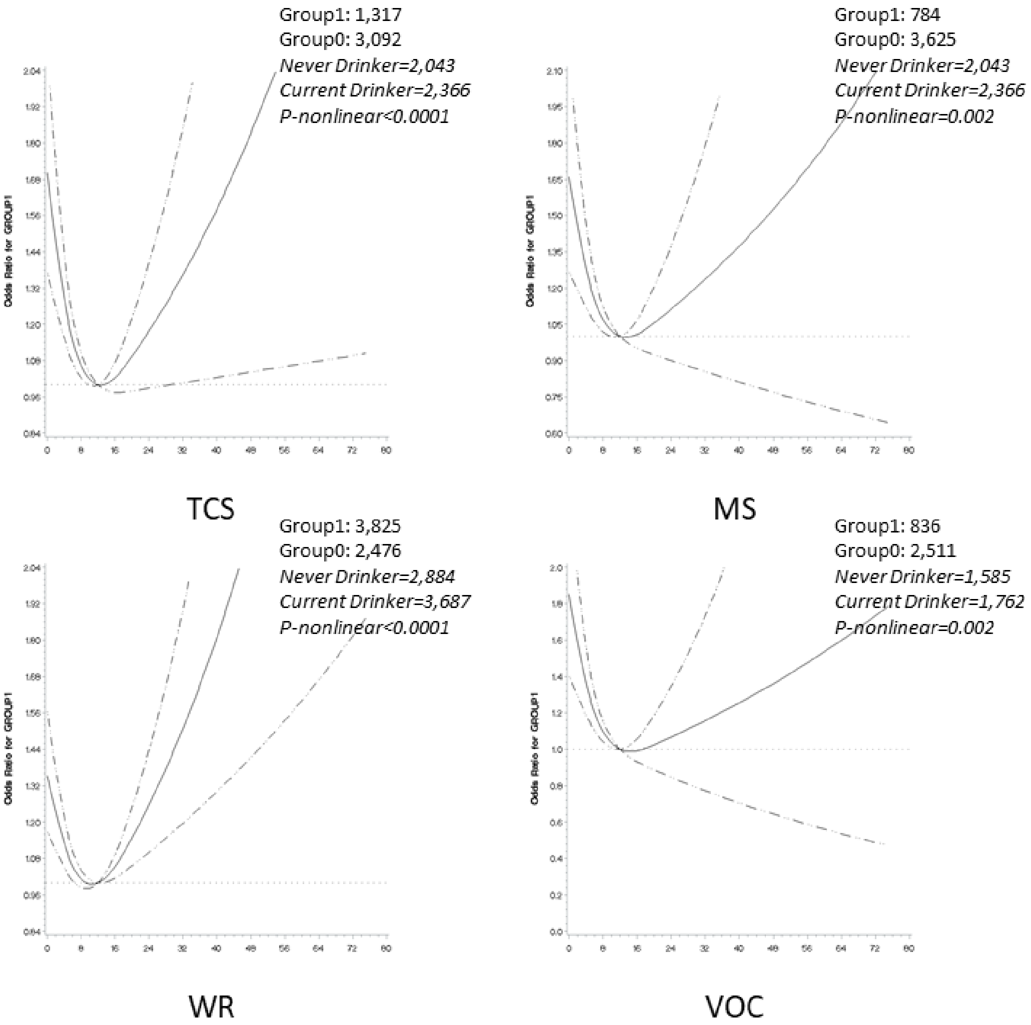


Note: Number of never drinker is 2043, 2043, 2884, and 1585; number of current drinker is 2366, 2366, 3687, and 1762 for TCS, MS, WR, and VOC, respectively.

© 2020 Zhang R et al. *JAMA Network Open*.

**eFigure 7. Nonlinear Relationship Between Weekly Drinking Level and Cognition Trajectory Among Female Current and Never Drinkers**

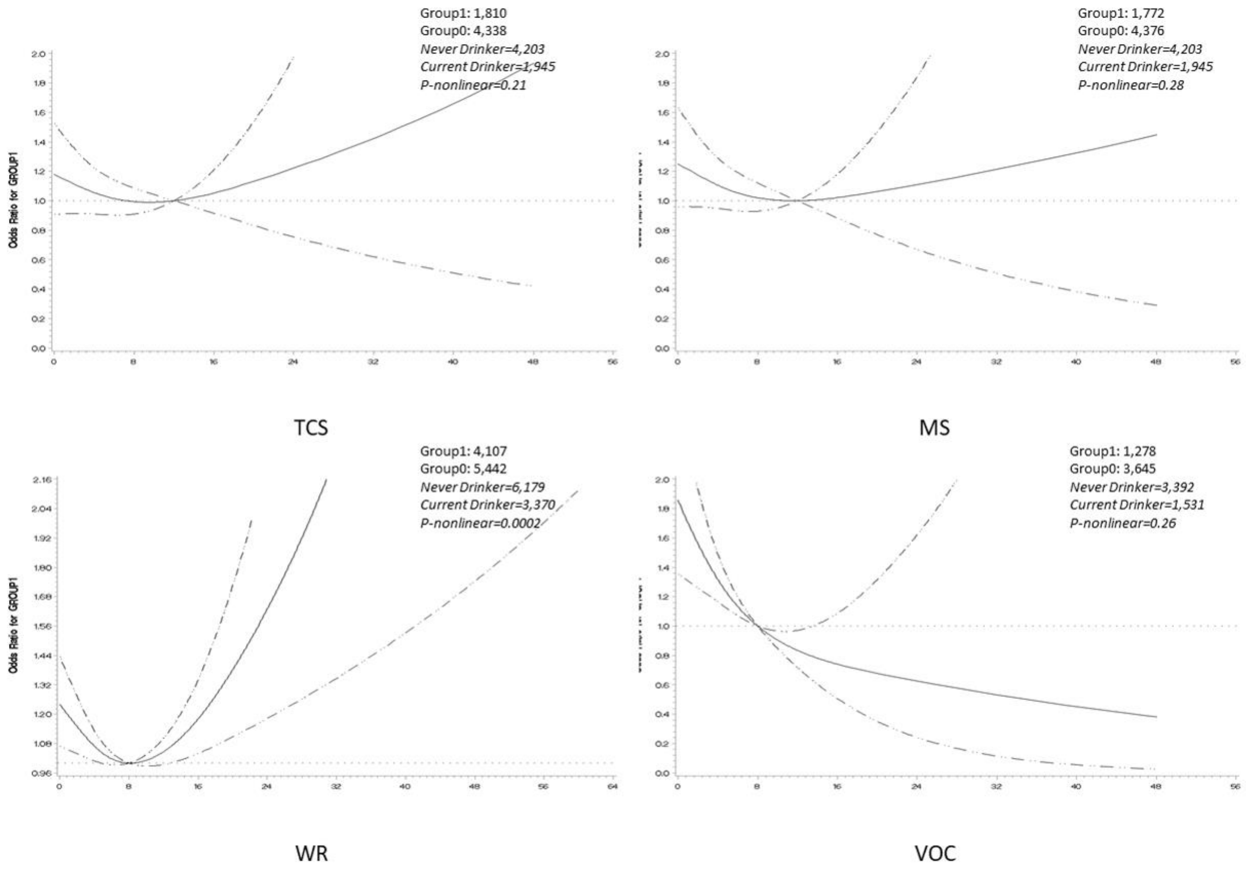


Note: Number of never drinker is 4203, 4203, 6179, and 3392; number of current drinker is 1945, 1945, 3370, and 1531 for TCS, MS, WR, and VOC, respectively.

© 2020 Zhang R et al. *JAMA Network Open*.

## eFigure 8. Nonlinear Relationship Between Weekly Drinking Level and Cognition Trajectory Among White Current and Never Drinkers

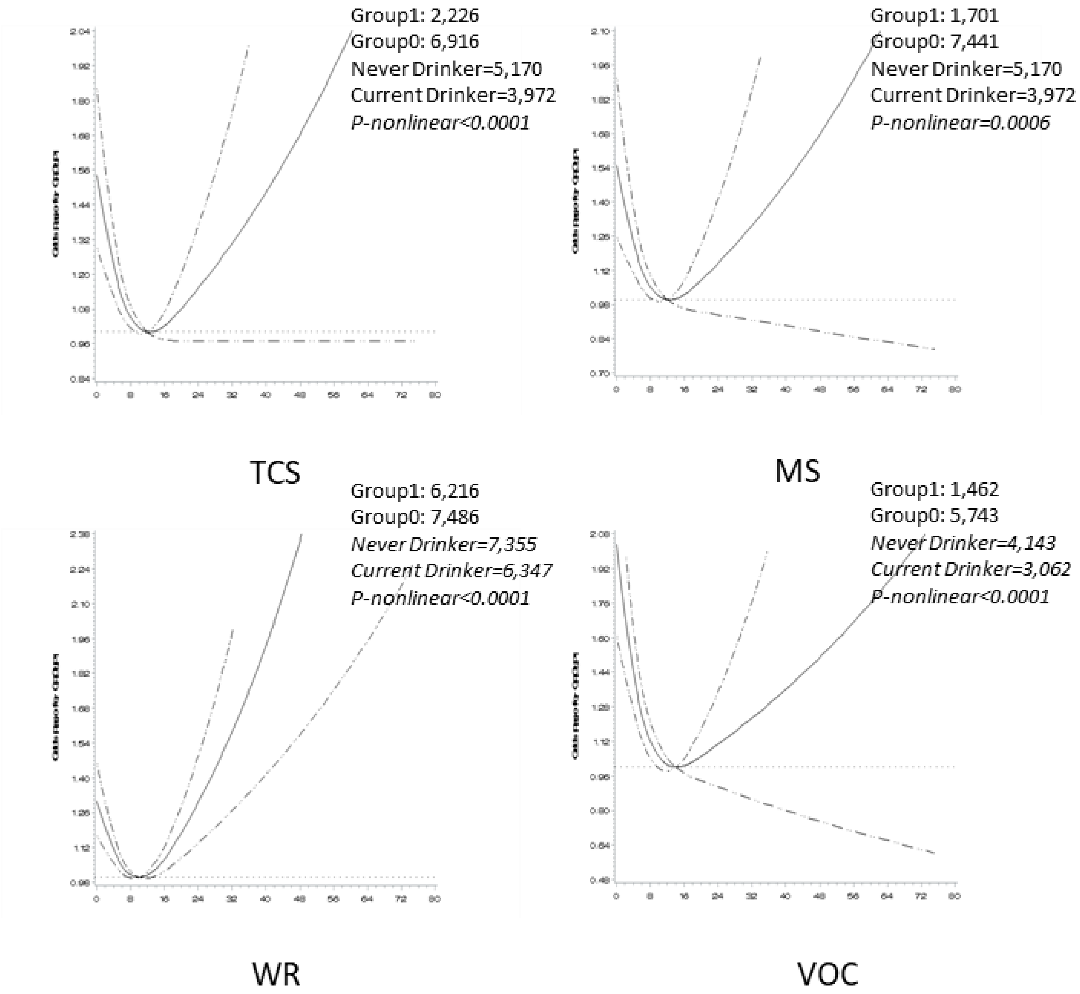


Note: Number of never drinker is 5170, 5170, 7355, and 4143; number of current drinker is 3972, 3972, 6347, and 3062 for TCS, MS, WR, and VOC, respectively.

© 2020 Zhang R et al. *JAMA Network Open*.

**eFigure 9. Nonlinear Relationship Between Weekly Drinking Level and Cognition Trajectory Among African American (AA) Current and Never Drinkers**

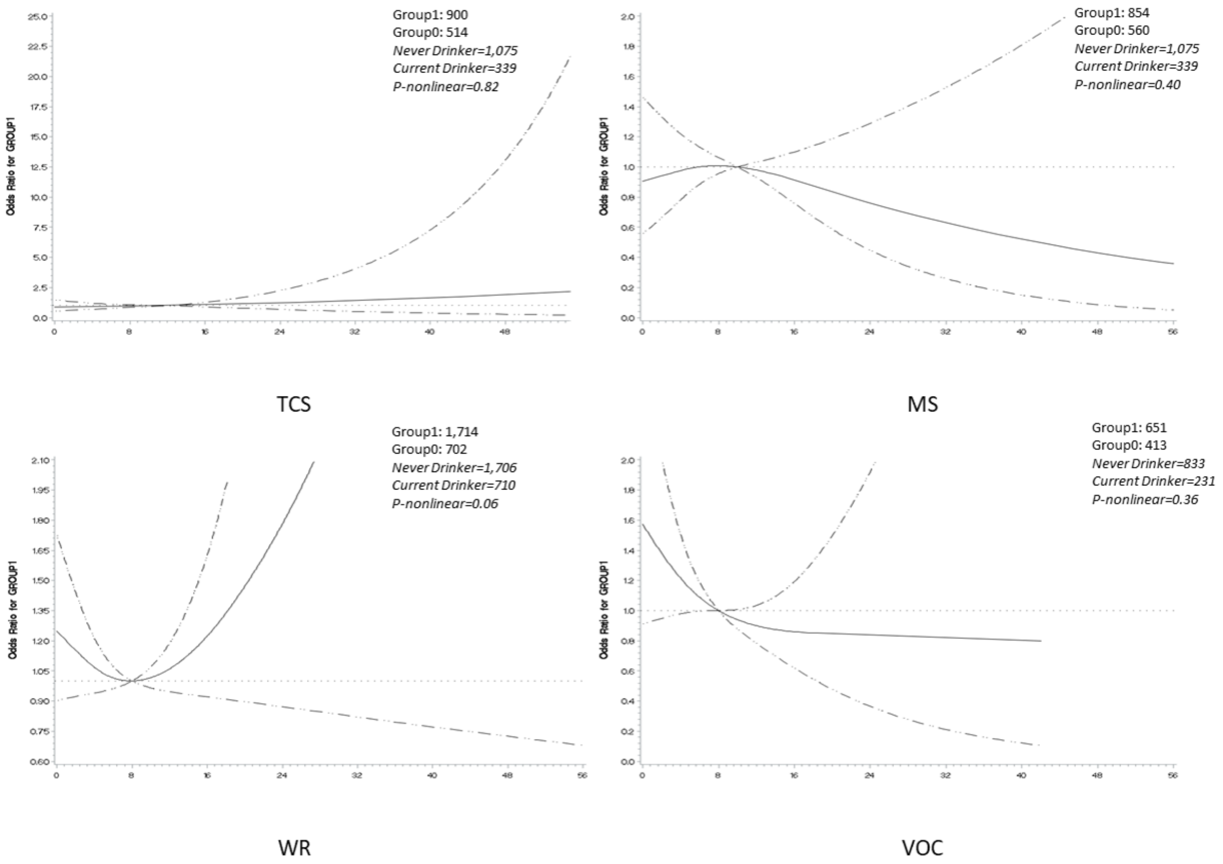


Note: Number of never drinker is 1075, 1075, 1706, and 833; number of current drinker is 339, 339, 710, and 231 for TCS, MS, WR, and VOC, respectively.

© 2020 Zhang R et al. *JAMA Network Open*.
